# Women’s contribution to stem cell research for osteoarthritis: an opinion paper

**DOI:** 10.3389/fcell.2023.1209047

**Published:** 2023-12-19

**Authors:** Émilie Velot, Elizabeth R. Balmayor, Lélia Bertoni, Susan Chubinskaya, Flavia Cicuttini, Laura de Girolamo, Magali Demoor, Brunella Grigolo, Elena Jones, Elizaveta Kon, Gina Lisignoli, Mary Murphy, Danièle Noël, Claire Vinatier, Gerjo J. V. M. van Osch, Magali Cucchiarini

**Affiliations:** ^1^ Laboratory of Molecular Engineering and Articular Physiopathology (IMoPA), French National Centre for Scientific Research, University of Lorraine, Nancy, France; ^2^ Experimental Orthopaedics and Trauma Surgery, Department of Orthopaedic, Trauma, and Reconstructive Surgery, RWTH Aachen University Hospital, Aachen, Germany; ^3^ Rehabilitation Medicine Research Center, Mayo Clinic, Rochester, MN, United States; ^4^ CIRALE, USC 957, BPLC, École Nationale Vétérinaire d'Alfort, Maisons-Alfort, France; ^5^ Rush University Medical Center, Chicago, IL, United States; ^6^ Musculoskeletal Unit, Monash University and Rheumatology, Alfred Hospital, Melbourne, VIC, Australia; ^7^ IRCCS Ospedale Galeazzi - Sant'Ambrogio, Orthopaedic Biotechnology Laboratory, Milan, Italy; ^8^ Normandie University, UNICAEN, BIOTARGEN, Caen, France; ^9^ IRCCS Istituto Ortopedico Rizzoli, Laboratorio RAMSES, Bologna, Italy; ^10^ Leeds Institute of Rheumatic and Musculoskeletal Medicine, Leeds, United Kingdom; ^11^ IRCCS Humanitas Research Hospital, Milan, Italy; ^12^ Department ofBiomedical Sciences, Humanitas University, Milan, Italy; ^13^ IRCCS Istituto Ortopedico Rizzoli, Laboratorio di Immunoreumatologia e Rigenerazione Tissutale, Bologna, Italy; ^14^ Regenerative Medicine Institute (REMEDI), School of Medicine, University of Galway, Galway, Ireland; ^15^ IRMB, University of Montpellier, Inserm, CHU Montpellier, Montpellier, France; ^16^ Nantes Université, Oniris, INSERM, Regenerative Medicine and Skeleton, Nantes, France; ^17^ Department of Orthopaedics and Sports Medicine and Department of Otorhinolaryngology, Department of Biomechanical Engineering, University Medical Center Rotterdam, Faculty of Mechanical, Maritime and Materials Engineering, Delft University of Technology, Delft, Netherlands; ^18^ Center of Experimental Orthopedics, Saarland University and Saarland University Medical Center, Homburg/Saar, Germany

**Keywords:** osteoarthritis, regenerative medicine, orthobiologics, stem cells, extracellular vesicles, gene therapy, RNA therapeutics

## 1 Introduction

The overall burden associated with musculoskeletal (MSK) conditions surpasses that of tobacco-related health effects, cancer, and diabetes (Barbour et al., 2017; United States Bone and Joint Initiative, 2020). Osteoarthritis (OA) is the most common degenerative joint disorder and a major worldwide challenge for health systems. OA is the leading cause of chronic disability among older adults (Anderson et al., 2011; Oo et al., 2021). It is a heterogenous condition with the pathogenesis differing across different joints. For example, obesity and meta-inflammatory processes have a significant role in knee OA while the shape of the joint and determinants of this are important in hip OA. Targeting the underlying pathological processes will be important in order to develop effective therapies. Post-traumatic OA (PTOA) develops after joint injury and is responsible for 12% of the cases of OA in the US. According to the US Bone and Joint Initiative (https://www.usbji.org), 62% of individuals with OA are women, being more prone to peripheral OA (hand, foot, knee) and experiencing more severe pain and disabling than men (Hawker, 2019; Leifer et al., 2022; Peshkova et al., 2022). Available pharmacological treatments only provide symptomatic pain relief and fail to arrest the progressive degeneration of cartilage associated with PTOA or idiopathic OA (Anderson et al., 2011; de Girolamo et al., 2016; de Girola et al., 2019). Surgical procedures that promote cartilage repair are expensive, require long rehabilitation, and might expose patients to the risk of complications. Thus, there is an imperative need to develop prophylactic novel biologic means that either prevent or treat early onset of the disease (Vincent, 2019; Vincent, 2020; Vincent et al., 2022).

The current management of OA is gradual and depends on the symptomatic evolution of the disease. Initially, a non-pharmacological approach can be proposed, mainly based on weight loss and the introduction of an adapted physical activity, accompanied by a general awareness of the patient to a healthy lifestyle (Skou and Roos, 2019). If insufficient symptom improvement is achieved with non-pharmacological approaches, pharmacological treatments using non-steroidal anti-inflammatory drugs (NSAIDs) are proposed to reduce pain and inflammation (Bannuru et al., 2019). Although their long-term efficacy and safety are still controversial, intra-articular injections of corticosteroids or hyaluronic acid, or a combination of both, are also being performed to improve the symptoms in patients who did not respond to conventional routes of analgesic administration (Fusco et al., 2021; Peck et al., 2021). Moreover, with the advent of orthobiologics, new potential therapeutic approaches are being exploited to preserve and, potentially restore, the articular surface (Kon et al., 2020; Kon and Di Matteo, 2021; Anzillotti et al., 2022). The group of S. Chubinskaya* has extensively studied the mechanisms of PTOA and the role of orthobiologics in cartilage repair and regeneration and successfully contributed to the clinical approval of the Agili-C implant (Anderson et al., 2011; de Girola et al., 2019). However, no drug can restore joint integrity or even stop the mechanisms leading to the development of OA and partial or total joint replacement by a prosthesis is considered for end-stage OA. Currently, 2,127 clinical trials related to OA (https://clinicaltrials.gov, accessed on 15 April 2023 under “OA+ drugs + interventional + female”) are underway to identify disease-modifying OA drugs (DMOADs) (Dietz et al., 2021; Kennedy et al., 2022). Of those expected to have clinical value, sprifermin and lorecivivint (SM04690) recently entered phase II clinical trials. Still, despite encouraging preliminary results in preclinical models (Deshmukh et al., 2018; Deshmukh et al., 2019), these clinical trials showed mitigated results on the global population studied, with encouraging results obtained in one subtype of patients (Yazici et al., 2020; Li et al., 2021).

Women have had a significant role in the translation of stem cell studies to clinical trials including for OA. This required the development of measurement tools to assess progression of structural disease in OA. That cartilage loss can be detected in a valid and reproducible way over 2 years was first shown using magnetic resonance imaging (MRI) by Wluka *et al.* (Wluka et al., 2004). This method was shown to detect the clinically important outcomes of pain (Wluka et al., 2004) and knee replacement (Cicuttini et al., 2004), meaning that it was now possible to use this method for proof of concept trials in OA. This has led to the testing of stem cells in those at high risk of OA, such as knee PTOA, with favorable results (Wang et al., 2017). The trials in this area have been summarized in a number of systematic reviews (Gong et al., 2021). These have tended to find consistent evidence for a beneficial effect of intra-articular injections of stem cells on articular cartilage and subchondral bone, irrespective of the source or contents of the stem cells. However, among the trials there remains significant heterogeneity in the source and composition of the injected cells, small to modest sample sizes, and the potential for publication bias. The use of stem cells remains an exciting area of endeavor (Fortier, 2005), including for OA where there is a lack of effective therapies. However, more work is needed before such therapies can be recommended in the management of the pathology.

In parallel with ongoing human studies, stem cells have been employed in veterinary medicine (Fortier and Travis, 2011; Ferris et al., 2014; Colbath et al., 2020) and the European Drugs Regulations Agency already authorized the use of two products based on stem cells (Arti-Cell Forte^®^ and HorStem^®^) to treat mild to moderate signs of OA associated with lameness in horses, following various safety and efficacy studies of intra-articular injections (Bertoni et al., 2019; Broeckx et al., 2019; Be et al., 2021). As for humans, MSK injuries are the most prevalent and debilitating condition responsible for pain (in the form of lameness) and loss of performance associated with highly negative economic impact on the equine industry (IVIS, 2010). A wider range of therapies are used in equine practice compared with human practice to reduce the clinical signs and progression of OA (Velloso Alvarez et al., 2020), although there is still no curative treatment. In a “One Health, One Medicine” concept (Kahn, 2017), preclinical studies in horses led to applications in humans and *vice versa*, as recently illustrated by the marketing of the intra-articular polyacrylamide hydrogel (Arthrosamid^®^) or of autologous conditioned serum (ACS) (Camargo Garbin and Morris, 2021). Large animals including livestock, dogs or horses have similar biomechanical constraints to humans, leading to the development of MSK diseases in a similar pathogenesis (Yazici et al., 2020). Therefore, they are considered suitable target species and translational models for researching new therapeutic approaches, while small animal models are typically employed for investigating pathophysiological processes (McCoy, 2015; Manivong et al., 2023). Horses in particular demonstrated many similarities to human joints regarding articular cartilage thickness, cellular structure, biochemical composition, and mechanical properties. It is however interesting to note that in major international equestrian competitions and races, all genders compete in the same category and females achieve great victories (Hanousek et al., 2018).

**FIGURE 1:**
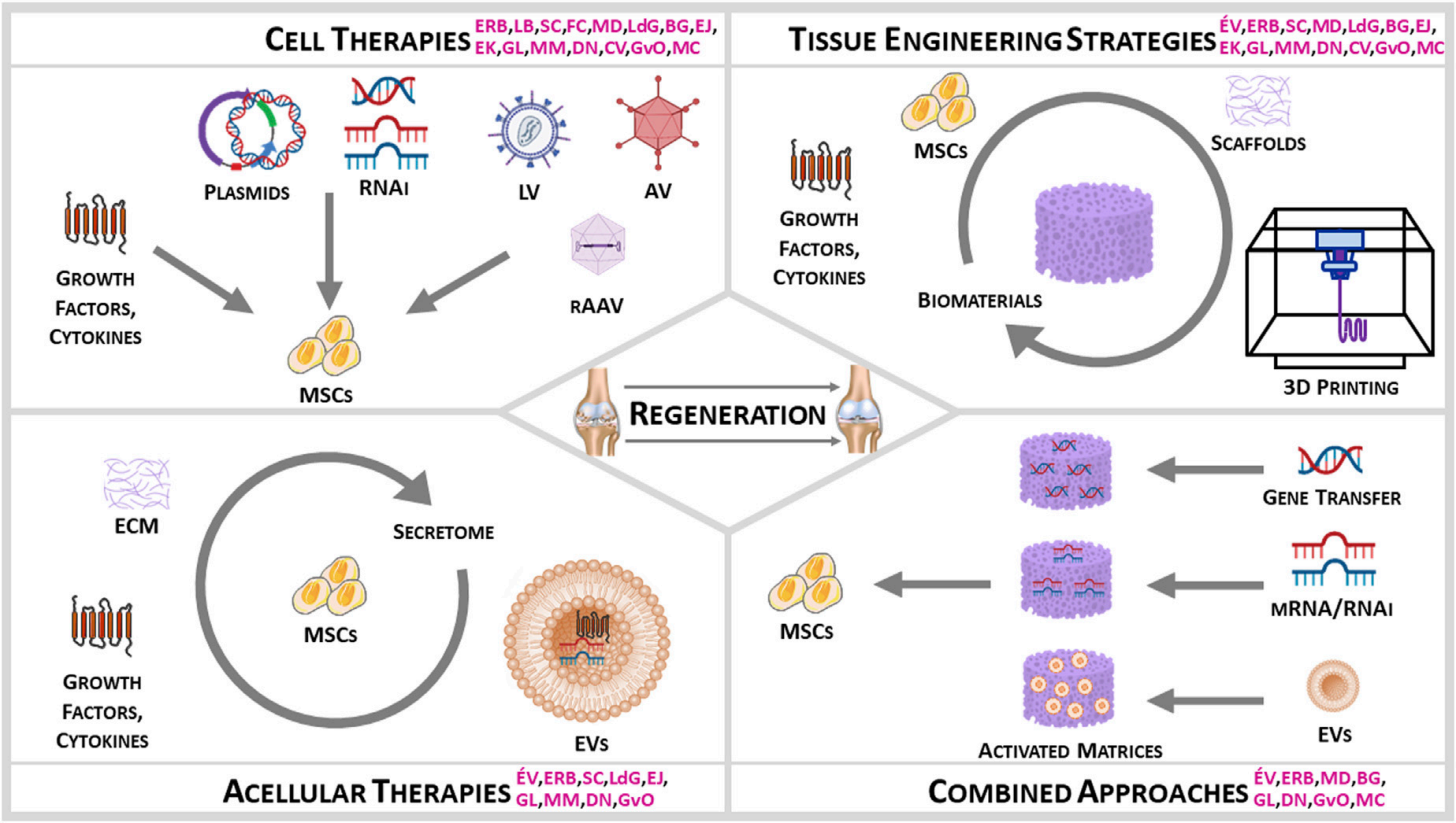
Women Input on Stem Cells for MSK Regeneration (initials in pink indicate the research contributions of the current authors).

This opinion paper focuses on the manipulation of mesenchymal progenitor cells as a promising therapeutic source of cells to treat MSK disorders with a focus on OA. This initiative was established by women researchers, with the purpose of highlighting some of the critical women’s contributions in the field of MSK tissue regeneration (including those of the current authors) as examples (Figure 1) with particular attention drawn in the text on outstanding women and on women (*) involved in the current opinion paper, and was performed under the umbrella of the International Cartilage Regeneration and Joint Preservation Society (ICRS; https://cartilage.org).

## 2 Cell-based therapies

### 2.1 Embryology and joint development, progenitor cells in the joint

The limb forms during embryogenesis as a limb bud, from the condensation of mesenchymal progenitor cells that chondrogenically differentiate into and form a cartilage anlage. This cartilage anlage will undergo endochondral ossification to form long bones, except at specific sites where an interzone is formed to give rise to the joint structures (Decker et al., 2014). The initial interzone consisting of growth and differentiation factor (*Gdf*)*5* ^+^ cells generated from descendants of chondrocytes which after cavitation will form the articular cartilage and intra-articular ligaments. Flanking cells recruited into the interzone from surrounding tissues also express *Gdf5* and will largely form the synovial lining and joint capsule. In adult mouse synovium, cells that originate from *Gdf5*-expressing joint interzone cells appear largely negative for the skeletal stem cell markers *Nestin*, Leptin receptor, and Gremlin1 (Roelofs et al., 2017), confirming a separate origin of these progenitor cells in synovium and skeletal progenitor cells. With different approaches, Decker *et al.* and Roelofs *et al.* demonstrated that upon osteochondral injury in adult mice, progenitor cells in the synovium proliferate and are recruited to the defect to contribute to tissue repair and articular chondrocytes have minimal contribution to tissue repair (Decker et al., 2017; Roelofs et al., 2017).

### 2.2 Joint MSC sources

Although initially referred to as stem cells, the definition of MSCs has been under discussion over the last decades (Viswanathan et al., 2019). The term “MSCs” is interchangeably used for Mesenchymal Stem Cells, Mesenchymal Stromal Cells, Medicinal Signaling Cells, or Multipotent Stromal Cells, and for the most part in connection to a potential clinical use. In this review, we use the term “MSCs” as such and the exact meaning refers to the context of their potential clinical use: either to generate tissue or to modulate tissue repair (Viswanathan et al., 2019). Distinct progenitor cells are present in the different joint tissues but it became clear that also within one tissue, different populations of progenitor cells exist. Generally, one can discriminate perivascular progenitor cell populations and populations located in the stroma or the lining. In the human synovium, the group of G. J. V. M. van Osch* (Sivasubramaniyan et al., 2019) described progenitor cells that are CD45^−^CD31^−^CD73^+^ and discriminated a CD90^−^ population in the intimal, synovial lining layer and a CD90^+^ population in the subintimal, perivascular area with distinct chondrogenic capacities. Similarly, synovial-derived MSCs have been reported in animal species by this group* (Teunissen et al., 2022). Moreover, as reported by this group in collaboration with the groups of E. Jones* (Sivasubramaniyan et al., 2018), in the human bone marrow, two different populations of CD45^−^CD271^+^ mesenchymal progenitor cells were found: a perivascular CD56^−^ population with poor chondrogenic capacity and a CD56^+^ bone lining population with good chondrogenic capacity. In bone marrow aspirations, the less chondrogenic perivascular population is more abundant than the bone lining population. A higher abundance of the more chondrogenic bone lining populations was found when the bone marrow was collected after rasping the marrow canal before implanting a prosthesis (Sivasubramaniyan et al., 2018). Bone marrow-derived (BM) MSCs have also been isolated from animals (Fortier et al., 1998; Kisiday et al., 2020). Thus, the location and way of collecting MSCs is an important determinant for their capacity to generate tissues.

Subcutaneous adipose tissue has gained popularity as a source of MSCs more recently compared with bone marrow. Nevertheless, many research efforts have addressed it as an optimal tissue source for a variety of clinical applications, as well as for an impressive number of basic studies to clarify MSC properties. Adipose tissue is indisputably a convenient tissue to harvest, with potentially no limit in terms of quantity and it contains a higher number of MSCs (adipose-derived stem cells, i.e., ASCs) with a similar immunophenotype to BM MSCs, bar the higher expression of CD34 seen in freshly isolated ASCs as shown by the groups of F. J. van Milligen and of D. Noël* (Varma et al., 2007; Maumus et al., 2013). Also, adipose tissue-derived MSCs have been reported by the group of H. Roelofs (Melie et al., 2013) to possess a higher immunomodulatory capacity than their bone marrow counterparts, making ASCs a more appealing cell type compared with BM MSCs in some clinical applications. ASCs are associated with the so-called stromal vascular fraction (SVF), a heterogeneous collection of cells composed of pre-adipocytes, endothelial precursor cells, T regulatory cells, macrophages, smooth muscle cells, and MSCs/pericytes indeed. Nevertheless, the SVF is a versatile cellular system and the degree of heterogeneity depends on a variety of factors, such as the adipose tissue harvest site, the digestion protocol, and the patient’s own pathological status. For this reason, there is no consensus on the exact definition and proportion of these cell types within the SVF. Also, culturing SVF cells for even one passage can profoundly alter their cellular composition as reported by Nunes *et al.* (Nunes et al., 2013) and observed by the group of D. Noël* (Domergue et al., 2016). From a therapeutic perspective, beyond exploiting the adipose tissue for the isolation and *in vitro* expansion of ASCs, the relatively higher abundance of MSCs within the adipose tissue has driven the development of several medical devices to isolate the SVF intra-operatively without the need for further cell expansion. These devices can either provide mechanical digestion of the adipose tissue resulting in a mechanical-SVF as reported by the group of Y. Kul (Tiryaki et al., 2022) and observed by the group of D. Noël* (Bony et al., 2016) or resize the adipose tissue into microfragments while removing oil and blood contaminants and resulting into microfragmented adipose tissue as shown by the group of L. de Girolamo* (Ulivi et al., 2022). Notably, in addition to the subcutaneous adipose tissue, other adipose sites have been investigated as MSC sources. In particular, in the MSK field ASCs isolated from the infrapatellar fat pad (or Hoffa’s body) have been demonstrated to possess a differentiation ability equal or even superior to subcutaneous ASCs (Lopa et al., 2014; Sun et al., 2018).

MSCs have additionally been found in healthy and OA synovial fluid (SF) by the group of E. Jones* (Jones et al., 2004; Jones et al., 2008) and in patients with chondral defects by G. J. V. M. van Osch* and K. Wright (Garcia et al., 2020). Compared with BM MSCs, SF MSCs are highly proliferative and consistently chondrogenic (Jones et al., 2008). In OA human knees, as shown in the work of the group of E. Jones* (Ilas et al., 2019; Sanjurjo-Rodriguez et al., 2019), subchondral BM MSCs are driven towards osteogenesis in order to rapidly compensate for load distribution alterations following the loss of cartilage. In contrast, they showed that SF MSCs from the same knees express higher levels of cartilage formation and turnover genes, and lower levels of ossification molecules (Sanjurjo-Rodriguez et al., 2020). Furthermore, SF MSCs display a pro-chondrogenic and immunomodulatory response following a joint-sparing, knee joint distraction procedure (Sanjurjo-Rodriguez et al., 2020). Owing to their apparent ability to respond to biomechanical cues, both types of joint-resident MSCs can be manipulated towards cartilage regeneration using mechanically-competent scaffolds and smart biomaterials.

MSCs have also been isolated from the articular cartilage by the group of L. A. Vonk (Rikkers et al., 2021; Rikkers et al., 2022) and peripheral blood (PB-MSCs), both showing a certain ability for osteochondrogenic differentiation (Mödder and Khosla, 2008; Beane and Darling, 2012; Martinello et al., 2013; Orth et al., 2014; Frisch et al., 2015a; Rikkers et al., 2021; Rikkers et al., 2022). Other sources of MSCs include perinatal MSCs from the umbilical cord blood or connective tissue or from the amniotic fluid and membrane (El Omar et al., 2014; Gómez-Leduc et al., 2016; Borghesi et al., 2019; Lepage et al., 2019; Hyland et al., 2020; Perry et al., 2021) as well as embryonic stem cells (ESCs) (Hwang et al., 2008), all with chondrogenic abilities and showing a higher potential for self-renewal and lower immunogenic and tumorigenic activities relative to adult MSCs. Of further note, as shown by the groups of É. Velot*, of J. Elisseeff, and of S. J. Kimber and by Lepage *et al.* and Borghesi *et al.* (Hwang et al., 2006; Oldershaw et al., 2010; El Omar et al., 2014; Dostert et al., 2017; Borghesi et al., 2019; Lepage et al., 2019; Mesure et al., 2019; Velot et al., 2021), MSCs from birth tissues have a better accessibility and can be non-invasively isolated in large amounts relative to adult MSCs while showing fewer ethical issues compared with ESCs.

The number of MSCs that can be obtained from a donor is limited. ESCs and induced pluripotent stem cells (iPSCs) represent a potentially unlimited source of chondrocytes. Protocols from ESCs have been adapted for iPSCs with slight variations. These stem cells can be used to specifically generate hypertrophic or non-hypertrophic chondrocytes as shown in particular by the groups of S. J. Kimber, of R. A. Kandel, of S. Diederichs, and of W. Richter (Oldershaw et al., 2010; Craft et al., 2013; Cheng et al., 2014; Diederichs and Tuan, 2014; Diederichs et al., 2019a). Non-hypertrophic chondrocytes generated from human ESCs have been reported to promote the repair of focal cartilage defects in rats (Cheng et al., 2014). Hypertrophic chondrocytes are generally generated from iPSCs by first differentiating them into iPSC-derived MSC-like progenitor cells (iMPCs). iMPCs generated from human BM MSCs were comparable to the parental MSCs, although iMPCs appear less responsive to traditional MSC differentiation protocols (Diederichs and Tuan, 2014). The considerable heterogeneity in chondrogenesis found using iMPCs has been associated with variable SRY-related high-mobility-group-box gene 9 (SOX9) protein expression, with low SOX9 levels correlating to high levels of the SOX9-antagonizing hsa-miR-145 (Diederichs et al., 2016). Still, the clinical use of iPSCs remains controversial since these cells are potentially tumorigenic and immunogenic, with an instability of their genome (Hackett and Fortier, 2011; Guzzo et al., 2013; Schnabel et al., 2014; Vonk et al., 2015; Driessen et al., 2017; Xu et al., 2019; Velot et al., 2021). However, the secretome of iPSC-derived MSCs (iMSCs) primed with tumor necrosis alpha (TNF-α) and interferon gamma (IFN-γ) has a high resemblance to BM MSCs, with iMSC-derived extracellular vesicles (EVs) showing similar *in vitro* immunomodulation (Ramos et al., 2022).

### 2.3 MSCs *versus* tissue-specific cells for joint regeneration

Murphy* *et al.* were the first to intra-articularly inject BM MSCs and study their potential for the treatment of OA using a caprine model (Murphy et al., 2003). Many studies in animals and in human patients followed. Since cell tracking studies showed only limited cartilage formation by these cells (Murphy et al., 2003; Hamilton et al., 2019; Markides et al., 2019; Perry et al., 2021), the mechanism of action was hypothesized to be related to factors secreted by the MSCs (Hamilton et al., 2019; Markides et al., 2019; Perry et al., 2021). Given their established immunomodulatory and trophic properties, MSCs isolated from different sources have been proposed as a viable therapeutic option to treat cartilage damage and early OA (Lopa et al., 2019). The group of D. Noël* noted the therapeutic efficiency of human and equine ASCs in the inflammatory model of collagenase-induced OA (Ter Huurne et al., 2012; Maumus et al., 2016). Intra-articular injection of immune-selected allogeneic human mesenchymal precursor cells were reported by the group of F. Cicuttini* to improve symptoms and structure in PTOA, suggesting that they modulate some of the pathological processes responsible for the onset and progression of this phenotype (Wang et al., 2017). These cells help to establish a “regenerative microenvironment” through the paracrine secretion of bioactive molecules and promote tissue-specific progenitor proliferation while inhibiting cell apoptosis and tissue fibrosis as reported by the group of L. de Girolamo* (Colombini et al., 2019). Among the molecules that play a role in the chondroprotective effect of MSCs in OA, Fra-1, THBS1 and TGFβi have been noted by the group of D. Noël* (Schwabe et al., 2016; Maumus et al., 2017; Ruiz et al., 2020). Alternatively to MSCs, tissue-specific cells have been also proposed as a valuable regenerative alternative for decades. Autologous Chondrocyte Implantation or Transplantation (ACI/ACT) was first developed in the 90s and since then has been used to treat thousands of patients with satisfactory results compared with other available techniques (Kon et al., 2013). Over time, a number of implementations of the original techniques have been developed, including the association of suitable biomaterials acting as scaffolds for cell growth and colonization (MACI, matrix-assisted autologous chondrocyte implantation) as shown by the large case series published by the group of E. Kon* (Kon et al., 2012). Nevertheless, while ACI/MACI resulted in the successful treatment of focal isolated chondral injuries, the clinical outcomes in patients with diffuse chondral damages are still under debate (Colombini et al., 2022a). One of the possibilities to make these techniques more effective in patients with diffuse cartilage defects (i.e., early OA patients) is to focus on chondroprogenitors. Chondroprogenitors are represented within cartilage as cells with migratory, clonogenic ability, and differentiation potential, found both in healthy and damaged cartilage as reported by Vinod *et al.* (Vinod et al., 2023). In addition to their direct stimulation within the cartilage, cartilage progenitors, likewise MSCs, could indeed be exploited to improve the existing cell-based therapies for the treatment of cartilage defects. In this regard, the possibility to enrich the amount of cartilage progenitors throughout *in vitro* expansion of the whole cartilage cell population seems a valuable option to improve the current results in early OA patients. It was observed that, with increasing passages in culture, cartilage cell populations are characterized by a progressive enhancement of clonogenic ability and sustained expression of stemness markers. Moreover, these expanded cells revealed a noteworthy chondrogenic potential, an enhanced secretory response to inflammation compared with MSCs, and strong immunomodulatory functions after inflammatory priming as seen by De Luca *et al.* (De Luca et al., 2019). Although data derived from animal models also show that chondroprogenitors have the ability to attenuate OA, repair, chondral defects, and form stable cartilage, displaying better outcomes than BM MSCs, these preclinical data would require further studies to optimize their use before clinical translation (Vinod et al., 2023).

Another possibility would be to combine the favorable properties of chondrocytes and MSC into a combined treatment. Apart from anti-inflammatory or anti-catabolic effects, the secretome of MSCs can also have trophic effects and in co-culture with chondrocytes, both adipose tissue-derived and BM MSCs demonstrated to enhance cartilage generation by the chondrocytes as reported by the group of G. J. V. M. van Osch* (Pleumeekers et al., 2018). This property of MSCs might explain the potential of a proposed one-stage cell therapy for cartilage defects where 75% of the chondrocytes can be replaced by MSCs (US20140329316A1, US20100144036A1), although the initial claim referring to an effect of the chondrocytes on the differentiation of the MSCs cannot be fully excluded.

### 2.4 Differentiation and culture conditions for appropriate MSK differentiation of MSCs, priming, immunomodulation

MSCs can be differentiated into cartilage-like cells *in vitro*. A large body of research from the groups of W. Richter and of G. J. V. M. van Osch* have investigated the stability of the cartilage formed by these cells and ways to improve this. They demonstrated that chondrogenically differentiated MSCs, though, were shown to be prone to endochondral ossification when implanted *in vivo* (Pelttari et al., 2006; Farrell et al., 2009). This finding redirected the bone tissue engineering field since more bone could be generated by pre-differentiating MSCs chondrogenically than the original attempts of pre-differentiating them towards the osteogenic lineage. Whereas research mostly focused on stimulating the chondrogenic capacity of these cells, such as, for example, by addition of parathyroid hormone-related protein (PTHrP) (Fischer et al., 2014; Fischer et al., 2016), TGFβi, NMB, or miR-29a as noted by the group of D. Noël* (Ruiz et al., 2020; Maumus et al., 2021; Guérit et al., 2014), inhibition of the anti-chondrogenic regulators became an emerging area (Lolli et al., 2019a). Inhibition of SMAD1/5/9 (Helli et al., 2011), WNT (Narcisi et al., 2015; Diederichs et al., 2019b), or anti-chondrogenic miRNAs, such as, for example, miRNA221 (Lolli et al., 2016) and miR-574–3p as observed by the group of D. Noël* (Guérit et al., 2013), has shown promise to inhibit hypertrophic differentiation. In addition to modulating chondrogenesis during the differentiation phase, modulation of the MSC expansion medium, for example, by the addition of WNT (Narcisi et al., 2015) or of TNF-α (Voskamp et al., 2020) resulted in improvement of chondrogenic capacity. The fact that these factors have demonstrated anti-chondrogenic properties during the differentiation phase demonstrates the complexity and importance of paying to the different stages of cell differentiation.

The immunomodulatory capacity of MSCs and ASCs can be influenced by disease status of the joint as noted by the groups of G. J. V. M. van Osch* and of G. Lisignoli* (Leijs et al., 2012; Manferdini et al., 2020). The immunomodulatory ability of ASCs was not affected by hypoxia as reported by the group of G. J. V. M. van Osch* (Roemeling-van Rhijn et al., 2013), whereas it was positively affected by an inflammatory stimulus such as interleukin 1 beta (IL-1β) as observed by the groups of L. de Girolamo* and of D. Noël* (Colombini et al., 2022b; Maumus et al., 2016). Similarly, the protein cargo of umbilical cord EVs was affected by their pro-inflammatory priming, but not in hypoxic conditions (Hyland et al., 2020).

### 2.5 Gene therapy

Stem cells are amenable to genetic modification to enhance their reparative potential in a variety of MSK disorders. Gene therapy is the transfer of candidate nucleic acid sequences as direct molecules or using a gene vector derived from nonviral material (most commonly a plasmid) or from viral material (adenoviral [AV], herpes simplex viral, retro-/lentiviral [LV], or recombinant adeno-associated viral [rAAV] vectors) to prolong the therapeutic effects of a gene product compared with recombinant agents with short pharmacological half-lives (Jorgensen et al., 2001; Noël et al., 2002; Jorgensen et al., 2003; Djouad et al., 2009; van Osch et al., 2009; Vinatier et al., 2009; Coleman et al., 2010; Ansboro et al., 2012; Cucchiarini et al., 2012; Demoor et al., 2014; Frisch et al., 2015a; Docheva et al., 2015; Balmayor, 2015; Cucchiarini, 2016; Fris et al., 2016; Jones et al., 2016; Rey-Rico and Cucchiarini, 2017; Poh et al., 2018; Mesure et al., 2019; Roseti et al., 2019; De la Vega et al., 2021; Amini et al., 2022). Gene therapy is performed *via* classical gene transfer or using genome editing methodologies such as use of the clustered regularly interspaced short palindromic repeats (CRIPSR)-associated 9 (CRISPR/Cas9) system (Tanikella et al., 2020). The contribution of women researchers in the field of gene therapy to target stem cells is broadly illustrated by reports showing the improved commitment of stem cell to MSK profiles using various classes of gene transfer vectors. Nonviral vectors carrying the SOX9 transcription factor were reported by the group of A. Rey-Rico to activate MSC chondrogenesis (Carball et al., 2022) (or the vascular endothelial growth factor - VEGF - to tackle MSC osteogenesis and bone healing as noted by the group of W. Richter (Geiger et al., 2007)). Adenoviral vectors carrying IL-10 were successfully used by the group of M. Murphy* to prevent OA (Farrell et al., 2016) (or bone morphogenetic proteins - BMP-2, BMP-6 - to modulate MSC osteogenesis and bone healing as reported by the group of A. L. Bertone (Zachos et al., 2006; Zachos et al., 2007; Murray et al., 2010; Ishihara et al., 2015)). rAAV vectors were manipulated by the groups of C. R. Chu, of L. R. Goodrich, and of M. Cucchiarini* to deliver the basic fibroblast growth factor (FGF-2) (Cucchiarini et al., 2005; Cucchiarini et al., 2011), transforming growth factor beta (TGF-β) (Pagnotto et al., 2007; Lee et al., 2011; Frisch et al., 2014a; Frisch et al., 2016; Frisch et al., 2017a; Cucchiarini et al., 2018), insulin-like growth factor I (IGF-I) alone (Frisch et al., 2014b; Cucchiarini and Madry, 2014; Frisch et al., 2015b; Frisch et al., 2017b) or with TGF-β (Morscheid et al., 2019a; Morscheid et al., 2019b), BMP-3 (Venkatesan et al., 2022), SOX9 (Cucchiarini et al., 2013), and chondromodulin (Klinger et al., 2011) to stimulate MSC chondrogenesis and cartilage repair (or BMP-2 for MSC osteogenesis (Ball et al., 2019)). Gene silencing in MSCs based on gene knockdown strategies using RNA interference (RNAi) technology is also a potent approach to modulate the reparative potential of these cells for MSK regeneration (Lolli et al., 2017; Demoor et al., 2014). For instance, silencing the antichondrogenic regulator microRNA 221 (miR-221) *via* specific antagomiR-221 or antimiR-221 oligonucleotides is capable of enhancing chondrogenesis and cartilage repair as seen in work from the group of G. J. V. M. van Osch* (Lolli et al., 2016; Lolli et al., 2019b).

### 2.6 Gene therapy modifications

Adenoviral- and rAAV-based vectors, while considered the most promising systems for OA, still have two main limitations: potential anti-viral host immune responses and the levels of efficiency and specificity of transgene expression in joint tissues. Targeting the joint cells of interest is still arduous even using intra-articular injections. Indeed, chondrocytes embedded in a dense extracellular matrix (ECM) are less accessible than synovial, ligament, or fat pad cells. In addition, disturbances in intracellular trafficking may decrease the efficiency of viral vector transduction. To address these issues, optimization of the carrier nucleic acid sequence and a modification of viral vectors have been considered with increasing interest. The capsids of adenoviral and rAAV vectors promote tropism but can also be recognized by neutralizing antibodies present in the circulation and synovial fluid as evidenced by the group of N. Bessis (Cottard et al., 2004) due to a pre-existing immunity, overall reducing the transduction efficiency. Yet, capsids may be engineered to escape such neutralization, and in this context, several improved hybrid AAV serotypes have emerged. Among them, the rAAV 2.5, a chimera of AAV1 and AAV2, evaluated in preclinical models of equine OA by Watson-Levings *et al.* (Watson Levings et al., 2018a) is currently being used in a clinical trial for OA (NCT05454566) and rAAV-DJ, which has been successfully used in a preclinical rat model of OA by Martinez-Redondo *et al.* (Martinez-Redondo et al., 2020). Also of interest in OA, Eichhoff *et al.* (Eichhoff et al., 2019) designed an rAAV preferentially targeting cells expressing the purinergic receptor (P2X_7_R) overexpressed in OA chondrocytes. Chemical modification allowing a large panel of chemical compounds or peptides to be coupled may also improve capsids to limit off-target effects, overcome undesirable properties, and permit viral escape from neutralizing antibodies. For instance, the chondrocyte-affinity peptide (CAP) has been used by the group of P. Pothacharoen (Chongchai et al., 2023) to enhance rAAV targeting of chondrocytes. Finally, self-complementary AAV (scAAV) composed of a double-stranded DNA genome have been successfully used by Watson-Levings *et al.* (Watson Levings et al., 2018a; Watson Levings et al., 2018b) to target chondrocytes, bypassing their step-limiting, low rates of cell replication/proliferation.

### 2.7 Transcript therapy

Despite the clear advantages of gene therapy, safety concerns related to viral vectors and affordability issues have restricted their clinical use as noted by the group of E. R. Balmayor* among others (Evans, 2019; De la Vega et al., 2021) In fact, affordability remains a pressuring issue, with gene therapy products tagged at $2 million a dose (Dyer, 2020) related to the manufacturing process under Good Manufacturing Practice (GMP). In addition, achieving efficient gene transfer and appropriate levels of transgene expression proved to be cumbersome for some applications as described by the group of E. R. Balmayor* (De la Vega et al., 2021), motivating researchers to explore alternatives to traditional MSK gene therapy. Applied to tissue regeneration, gene therapies commonly deliver protein-coding DNA. However, messenger RNA (mRNA), offers a more efficient way to achieve protein translation while accommodating numerous advantages as explained by Balmayor* *et al.* (Balmayor, 2022). Like traditional gene therapy, mRNA transcript therapy offers control over the production of therapeutic proteins. In addition, since mRNA function is conducted in the cell cytoplasm, it results in an immediate initiation of translation. Unlike viral gene therapy, mRNA transcript therapy is safe as it does not carry the risk of insertional mutagenesis and oncogenesis. Also, mRNA’s transient nature is valuable in many regenerative medicine applications when long-term effects are not necessary as stated by others and by Balmayor* *et al.* (Sahin et al., 2014; Balmayor and Evans, 2019). mRNA is produced by *in vitro* transcription (IVT), a relatively simple, single-reaction procedure easily controllable and scalable and identical for all mRNAs, regardless of the sequence. Importantly, no cell or bacterial culture is required, reducing the risk of contamination. IVT allows the introduction of numerous chemical modifications and the use of modified nucleosides to produce defined mRNAs with reduced immunogenicity and low stability, with costs described as up to 10-fold lower that their protein therapeutic counterparts as demonstrated by K. Karikó among others (Karikó et al., 2005; Weissman, 2015). The recent use of mRNA in COVID-19 vaccines receiving approval in various countries demonstrates that this technology can be safe and efficient. Regarding MSK regeneration, local mRNA delivery has been investigated mostly for bone by the group of E. R. Balmayor* and by Badieyan *et al.* and Khorsand *et al.* (Elangovan et al., 2015; Badieyan et al., 2016; Balmayor et al., 2016; Balmayor et al., 2017; Khorsand et al., 2017; Zhang et al., 2019; Fayed et al., 2021; Geng et al., 2021; De La Vega et al., 2022; European Scoiety of Gene and Cell Therapy, 2022), with a strong focus on effective BMP-2 mRNAs but also with an emerging interest for BMP-9 (Khorsand et al., 2017), BMP-7 (European Scoiety of Gene and Cell Therapy, 2022), and VEGF mRNAs by Geng *et al.* (Geng et al., 2021). In one of the most translatable studies performed to date, a BMP-2 mRNA administered to rat femoral critical-size defects *via* a collagen carrier was reported by the group of E. R. Balmayor* (De La Vega et al., 2022) to remain local in a safe manner and to efficiently induce bone healing in a dose-dependent manner. For cartilage repair, mRNAs coding for the runt-related transcription factor 1 (Runx1) (Aini et al., 2016), Link N by the group of M. Avci-Adali (Tendulkar et al., 2019), and IGF-I (Wu et al., 2022) have been investigated as OA treatments. Direct injections of Runx1 mRNA in mouse OA knee joints significantly suppressed OA progression (Aini et al., 2016) while injection of stem cells transfected with an IGF-I mRNA in mouse knee joints safely enhanced cell survival and engraftment in the cartilage tissue (Wu et al., 2022). Overall, all these findings support the safety and efficacy of this emerging technology for MSK regeneration.

## 3 Tissue engineering strategies

### 3.1 Tissue engineering

New tissue engineering therapeutic strategies mainly based on the use of biomaterials combined with stem cells showed good potential both in preclinical and clinical studies as reported by the groups of G. Lisignoli*, of D. Noel* and of L. R. Goodrich and by Nooeaid *et al.*, Moradi *et al.*, Taraballi *et al.*, Armiento *et al.*, and Kwon *et al.* (Nooeaid et al., 2012; Moradi et al., 2017; Taraballi et al., 2017; Yang et al., 2017; Armiento et al., 2018; Kwon et al., 2019; Pascual-Garrido et al., 2019; Trucco et al., 2021, Valot et al., 2021; Manferdini et al., 2022). Regenerative scaffold-based methods are emerging as possible therapeutic alternatives for the treatment of various types of cartilage lesions as noted by the group of E. Kon* (Kon et al., 2015). In order to support the proliferation of live cells, a scaffold is a temporary three-dimensional (3D) framework made of biodegradable polymers that may replicate the highly structured functional architecture of the articular cartilage. Chondrogenic differentiation of MSCs can be achieved on different types of scaffolds provided that a growth factor can be released to induce the process as observed by the group of D. Noël* (Morille et al., 2013; Morille et al., 2016; Mathieu et al., 2014). Different types of chondral and osteochondral scaffolds have been used in the past, but the most promising results have being obtained with biodegradable scaffolds as observed by the group of E. Kon* (Kon et al., 2014; Kon et al., 2018; Kon et al., 2021; Altschuler et al., 2023). The use of scaffolds is often integrated with cells seeded on the scaffold itself. The group of E. Kon* (Kon et al., 2008; Kon et al., 2012) was the first to use articular chondrocytes in association with biomaterials for cartilage regeneration in clinical practice with discrete success in treating focal defects. Although some products containing chondrocytes and biomaterials are still on the market, the necessity of addressing different tissues and the need of modulating the joint environment pushed towards the use of stem cells instead of chondrocytes.

Both natural and synthetic biomaterials have been used to regenerate different joint tissues (cartilage, bone, meniscus) and cells have been mainly seeded or encapsulated into these structures for instance by Sanchez-Tellez *et al.*, Yang *et al.*, and Critchley *et al.* (Sánchez-Téllez et al., 2017; Li et al., 2019; Yang et al., 2019; Critchley et al., 2020; Rahman et al., 2022). However, the main limitation of this approach is the retention of stem cells in the target joint tissue that may be influenced by the presence of synovial fluid, inflammation, load, and joint movement as noted by the groups of E. Kon* and of G. J. V. M. van Osch* and by Bakhshandeh *et al.* (Fahy et al., 2014; Perdisa et al., 2014; Bakhshandeh et al., 2017; Dong et al., 2017). To overcome this issue, it is necessary to design advanced biomaterials capable of retaining cells *in situ*, with immunomodulating properties while at the same time, retaining the capacity to stably adhere to damaged joint tissues and boost their regeneration. Biomaterial stiffness and viscoelasticity, as other critical parameters, are selected depending on the anatomical characteristics of the regenerated joint tissues as reported for instance by Sarem *et al.* (Sarem et al., 2018; Zhang et al., 2020; Huang et al., 2023). All biomaterial parameters directly control and influence the characteristics of the cells by acting through specific receptors directing their migration, proliferation, matrix synthesis, and differentiation as observed by the group of G. Lisignoli* and by Yang *et al.*, Sarem *et al.*, and Hafezi *et al.* (Sarem et al., 2018; Yang et al., 2019; Hafezi et al., 2021; Sartore et al., 2021). These strategies contribute to joint tissue regeneration by showing effects on different physiological processes including inflammation, metabolic processes, aging, apoptosis, and autophagy as shown by the groups of E. R. Balmayor* and of G. Lisignoli* and by Koh *et al.* (Amann et al., 2017; Chen et al., 2020; Koh et al., 2020; Gabusi et al., 2022).

Stem cells have also been reported to secrete an ECM that may be valuable for MSK regeneration. For instance, MSCs from the connective tissue of the umbilical cord (Wharton’s jelly) produce ECM components similar to those of articular cartilage as reported by Russo *et al.* (Russo et al., 2022) and participate in MSC chondrogenic differentiation, offering a potential scaffold that may be used as an alternative to improve cartilage regeneration as noted by Ramzan *et al.* (Ramzan et al., 2022).

### 3.2 Bioprinting, sustainable automated manufacturing for therapeutic cells

Emerging technologies like additive manufacturing (three-dimensional [3D] printing) will lead future directions for tissue engineering therapeutic strategies. 3D printing replicates the damaged tissue shape starting from the patient medical image. It consists of the fabrication of living tissue/organ-like structures throughout the bottom-up deposition of either cell-laden droplets or cells embedded in a hydrogel, in both cases termed as “bioink” as described for instance by Abdollahiyan *et al.* (Abdollahiyan et al., 2020). Such a technology makes is possible to overcome issues associated with more conventional methods like static (manual) seeding onto scaffolds or dynamic seeding using bioreactors as shown by the group of B. Grigolo* (Roseti et al., 2018). In these cases, problems are due to cell accumulation at the surface of scaffold and to the low density in the inner part where cells tend to die because of the scarcity of nutrients. This may lead to inaccurate experimental results and consequent speculation. Differently, 3D bioprinting offers the advantage of fine control of cell spatial distribution in terms of homogeneity. When scaffolds were cultured with chondrogenic or osteogenic medium, cartilage and bone tissues were produced, respectively, as determined by specific gene and protein expression (Gao et al., 2015). Further benefits of 3D bioprinting techniques include reduced production times, an increased versatility, and the possibility to work at room temperature and “solvent-free” conditions, taking advantage of the features of water-based gels such as bioinks as reviewed by the groups of B. Grigolo* and of D. Noël* (Roseti et al., 2017; Montheil et al., 2022). 3D bioprinting also enables the fabrication of custom-made products based on patient’s medical images. Such options improve the match between implant and defect size, thus shortening the time required for surgery and for patient recovery, and positively affecting the success of treatment.

Development and use of bio-inspired, fabricated bio-printed constructs will require validated and relevant cell sources and/or use of appropriate factors to attract endogenous stem cells to the constructs. The cost of producing GMP-grade MSCs, or even iMSCs, using manual processes is such that translation to patient use at scale to enable widespread use is prohibitive and a critical impediment in the field as noted by the group of M. Murphy* (Ochs et al., 2022). The automation of production of advanced therapy medicinal products (ATMPs), state-of-the-art medicines for human use based on genes, tissues or cell-derived EVs can address this hurdle to clinical translation as reported by the group of M. Murphy* (Ochs et al., 2022).

### 3.3 From biologic substitutes to organoids, 3D models, and organ-on-a-chip

In accordance with the 3Rs concept (replacement, reduction, refinement) and with the strategic directions of the European Medicines Agency (EMA) (announcement of 29 September 2021) to promote alternative approaches to animal models, the development and use of effective alternative *in vitro* methods before formal animal testing are now mandatory. Advanced techniques such as biomanufacturing (3D bioprinting and bioassembly) and organotypic models (organoids, organ-on-a-chip) are gaining interest due to their ability to mimic OA conditions. Although 2D and 3D cellular cultures have limitations, they are still useful in answering fundamental questions in a molecular, cellular, and tissue continuum and provide a cost-effective platform for rapid, high-throughput screening of new drugs, delivery systems, or biolubricants. This provides an efficient standardized functional qualification without the use of experimental animals for more ethical research (Manivong et al., 2023). Tissue engineering studies conducted since the emergence of ACT in 1994 by Brittberg (Brittberg et al., 1994) led to the development of suitable biological substitutes and refinement of organotypic models by the group of M. Demoor* (Demoor et al., 2014). These mini-tissues/organoids have been incorporated in some translational research studies by the same group* (Desancé et al., 2018; Cullier et al., 2022) to develop cell-based and cell-free orthobiological therapeutic approaches. Therefore, some *in vitro* studies have been transposed from humans (Legendre et al., 2013; Gómez-Leduc et al., 2016; Gómez-Leduc et al., 2017; Legendre et al., 2017) to horses (Desancé et al., 2018) again by this group* to demonstrate the therapeutic potential of chondrocytes and stem cells for cartilage engineering in both species and to have the possibility to carry out preparatory work in horses before transposing it to humans.

## 4 Acellular therapies

### 4.1 Secretome

MSC capacity to induce tissue regeneration is due to its secretome that comprises all cell secretions (growth factors, cytokines, EVs, etc.). Among regenerative medicine strategies, acellular or cell-free therapies requires the delivery of exogenous active molecules, such as synthetic molecules or MSC secretome, as therapeutic agents into the joint as reported by Velot* *et al.* (Dostert et al., 2017; Velot et al., 2021).

Indeed, the secretome of BM MSCs that were primed with inflammatory factors were shown to inhibit inflammatory processes in human OA synovial explants *in vitro* as observed by the group of G. J. V. M. van Osch* (van Buul et al., 2012). The activity of the secretome of MSCs is not specific for BM MSCs (Manferdini et al., 2020). The groups of G. Lisignoli* and of D. Noël* showed that adipose tissue-derived MSCs (ASCs) have similar effects *in vitro,* and their anti-inflammatory and anti-catabolic effects were indicated to be dependent on the inflammatory status of the chondrocytes or synoviocytes (Maumus et al., 2016). The secretome of human BM MSCs reduced pain and joint degeneration in a mouse model of OA (Khatab et al., 2018).

Most animal studies aimed at improving acellular MSC-based therapies, especially in horses, are still predominantly conducted *in vitro* as reported by Jammes *et al.* (Jammes et al., 2023). The same author participated in a study (Contentin et al., 2022) that specifically highlighted the pro-anabolic potential of equine MSC-conditioned media containing exosomes on a cartilage organoid model *in vitro*. Kearney *et al.* (Kearney et al., 2022) performed a recent study in acute inflammatory arthritis by injection of lipopolysaccharide (LPS) in horses, showing no difference between the injection of allogenic BM MSCs and of their secretome. Additionally, proofs of concept for the manufacturing of clinical-grade equine and canine freeze-dried secretome which present many advantages in terms of practical use (stability, easy to store and use) have been published by Mocchi *et al.* (Mocchi et al., 2021a; Mocchi et al., 2021b). These authors showed in a canine study that an intra-articular injection of secretome in five dogs with OA was safe, without significant adverse effects. These preliminary data suggest that clinical trials may be established to evaluate the potential of these therapies in the treatment of spontaneous OA in animals.

### 4.2 Extracellular vesicles

Research is also well underway in animals to characterize the MSC secretome in order to identify the most promising factors for treating OA and to explore the EVs as potential biomarkers for monitoring its progression (Anderson et al., 2022). In particular, techniques for isolating and characterizing EVs have been described for several types of MSCs and work on chondrocytes indicated that MSC-EVs reduce gene expression of inflammatory markers (Hotham et al., 2021; Arévalo-Turrubiarte et al., 2022).

MSCs produce large and small size EV subtypes that exert similar protective effects on chondrocytes and anti-inflammatory effects on macrophages *in vitro* as reported by Vonk *et al.* and *in vivo* in the collagenase-induced OA model of the group of D. Noël* (Cosenza et al., 2017; Vonk et al., 2018). However, small size EVs were more potent to suppress the clinical signs of inflammatory arthritis in the collagen-induced model of the group of D. Noël* (Cosenza et al., 2018). This observation was attributed to the induction of a regulatory response by small size EVs as shown by the upregulation of CD19^+^IL10^+^ Breg-like cells and the decrease in plasmablast cells in the lymph nodes of mice. Down-regulating the TGF-β-induced gene product-h3 (TGFBI/BIGH3) in MSCs partly inhibited their anabolic effects on chondrocytes and did not protect mice from developing OA. Although not demonstrated to be directly related, the detection of TGFBI/BIGH3 in the cargo of both small and large size EVs might explain the protective role of EVs in OA as seen by the group of D. Noël* (Ruiz et al., 2020). Priming MSCs was shown to modify their proteome and secretome as well as their content in miRNAs, thereby modulating their functional properties, in particular their immunoregulatory function as described by the group of D. Noël* (Pers et al., 2021). Parental cell priming also impacts the cargo of EVs and inflammatory priming was reported to modulate cytokine and miRNA levels in EVs and enhance their anti-inflammatory potency (Ragni et al., 2020). Another strategy is based on genetic engineering of MSCs to overexpress the hypoxia-inducible factor 1 alpha (HIF-1α) and telomerase to generate large-scale production of reproducible batches of MSC-derived EVs with higher immunosuppressive activity (Gómez-Ferrer et al., 2021). In addition, overexpression of several miRNAs in MSCs or in synovial fibroblasts, as shown with miR-126–3p, may suppress apoptosis and inflammation in chondrocytes and prevent cartilage degradation in a OA model (Zhou et al., 2021).

Indeed, miRNAs were shown to be a crucial player for MSC-EVs function. This paradigm has been confirmed for EVs released by MSCs obtained from several sources such as adipose tissue, bone marrow, and amniotic membrane as studied by the group of L. de Girolamo* (Ragni et al., 2020; Ragni et al., 2021a; Ragni et al., 2022a). With respect to EV-miRNA activity for orthopedic conditions, a complex scenario has emerged with respect to how they can function/contribute to MSC therapeutic potential. MSC-EVs are enriched in miRNAs, predicted to suppress the activation of immune cells and the production of OA-related inflammatory mediators, as well as to promote cartilage protection by acting on both chondrocyte homeostasis and extracellular matrix-degrading enzymes. Most importantly, pre-activation of MSCs with pro-inflammatory mediators allows for the release of EVs with increased amounts of protective miRNAs, as clearly shown for adipose MSCs (Ragni et al., 2020). These results were confirmed by the group of L. de Girolamo* (Ragni et al., 2021b; Ragni et al., 2022b) for EVs released from MSCs in the presence of synovial fluid from OA patients that is rich in pro-inflammatory molecules. Consistently, when envisioned as a cell-free therapy, MSC-EVs protected mice from developing OA, suggesting that these nanoparticles can reproduce the main therapeutic effects of secreting cells by reducing OA symptoms (Cosenza et al., 2017). Notably, inhibition of inflammation (Cosenza et al., 2018) and reduction of cartilage degradation (Cosenza et al., 2017) *in vivo* confirmed *in silico* molecular data on EV-miRNAs fingerprints, leading to the potential for new strategies using single miRNA modulation (Xi et al., 2021) as a means to improve therapeutic potential for both MSK disorders and other diseases where cutting-edge treatments are actively needed.

Native EVs or EVs from primed cells do not necessarily contain appropriate mediators to improve OA. The use of synthetic vesicles such as liposomes may be a means to encapsulate and deliver exogenous active molecules of interest to joint tissues via intra-articular injection. This drug delivery system may counteract the degradation of selected mediators and allow for their sustained release in the joint (Velot et al., 2022). However, liposomes can be silenced by immune cell phagocytosis. As the composition of EV membrane prevents phagocytosis, new delivery systems to carry selected active molecules may be developed by merging MSC-derived EVs and liposomes to engineer a hybrid vesicle designed for joint healing as reported by Velot* *et al.* and by others (Velot et al., 2021; Elkhoury et al., 2022).

## 5 Combined approaches

### 5.1 Gene-activated matrices

MSK gene therapy has been further expanded by combining gene transfer technologies with tissue engineering to generate gene-activated matrices (GAMs), a powerful tool also referred to as biomaterial-guided gene therapy, improving the spatiotemporal, controlled delivery of therapeutic genes to their targets (Borghesi et al., 2019; Perry et al., 2021; Hyland et al., 2020; Hwang et al., 2008; Karikó et al., 2005; Weissman, 2015; Elangovan et al., 2015; Balmayor et al., 2016; Badieyan et al., 2016; Balmayor et al., 2017; Khorsand et al., 2017; Zhang et al., 2019; Geng et al., 2021; Fayed et al., 2021; De La Vega et al., 2022; European Scoiety of Gene and Cell Therapy, 2022; Aini et al., 2016; Tendulkar et al., 2019; Liu et al., 2022; Madry et al., 2020a; Venkatesan et al., 2019; Cucchiarini and Madry, 2019; Atasoy-Zeybek and Kose, 2018; Shapiro et al., 2018; Venkatesan et al., 2018; Rey-Rico et al., 2017a; D’Mello et al., 2017; Rey-Rico et al., 2016; Raisin et al., 2016; Cucchiarini et al., 2016; Raftery et al., 2016; Rey-Rico and Cucchiarini, 2016). Different systems (micelles, hydrogels, solid scaffolds) have been used to generate GAMs capable of delivering a variety of gene transfer vectors. Nonviral vectors were incorporated in GAMs by the groups of A. L. Bertone, of W. Richter, and of M. Murphy* and also by Curtin *et al.*, Raftery *et al.*, and Tierney *et al.* to carry PTH(1–34) (collagen) (Backstrom et al., 2004), VEGF (collagen) (Geiger et al., 2005), BMP-2 (collagen, nano-hydroxyapatite - nHA, alginate, chondroitin sulfate) (Curtin et al., 2012; Loozen et al., 2013; Nedorubova et al., 2022; Husteden et al., 2023), BMP-2/VEGF (collagen, nHA, chitosan) (Curtin et al., 2015; Raftery et al., 2017; Raftery et al., 2019; Walsh et al., 2021), BMP-2/BMP-7 (collagen, nHA, chitosan) (Raftery et al., 2018), SOX9 (collagen, alginate) (Ledo et al., 2020), ephrinB2 (collagen, nHA) (Tierney et al., 2013), and the stromal-derived factor 1 alpha (SDF-1α) (collagen, nHA) (Power et al., 2022) to stimulate MSC chondro-/osteogenesis and bone healing. GAMs formulating rAAV vectors carrying TGF-β (pluronics, carbon dots - CDs, poly (ε-caprolactone) - PCL) (Rey-Rico et al., 2017b; Meng et al., 2020; Venkatesan et al., 2021), IGF-I (alginate) (Maihöfer et al., 2021), and SOX9 (pluronics, PCL, CDs) (Rey-Rico et al., 2018; Madry et al., 2020b; Urich et al., 2020; Venkatesan et al., 2020) were also used by the group of M. Cucchiarini* to stimulate MSC chondrogenesis, cartilage repair, and prevent OA. Interestingly, GAM have been also tested by the groups of E. R. Balmayor*, of M. Murphy*, and of D. Noël* to deliver RNAs, like for instance an mRNA for BMP-2 (micro-macro biphasic calcium phosphate - MBCP - granules, fibrin gel, collagen sponge, titanium implants) (Tierney et al., 2013; Morille et al., 2016; Balmayor et al., 2017; Zhang et al., 2019; Fayed et al., 2021; De La Vega et al., 2022) to stimulate MSC osteogenesis and bone healing or siRNAs against Runx2 (pluronics, collagen) (Raisin et al., 2017; Salvador et al., 2022) to reduce MSC osteogenesis.

### 5.2 EVs and biomaterials

The use of MSC-derived EVs in combination with biomaterials represent a future MSK tissue engineering strategy to influence and facilitate local therapeutic release of EVs by means of their design and chemical characteristics (Yan et al., 2020; Casanova et al., 2021; Zhang et al., 2021). Nanofibrous substrates designed to immobilize EVs induce the chondrogenic differentiation of BM MSCs as seen by Casanova *et al.* (Casanova et al., 2021). Hydrogels containing MSC-derived EVs have the ability to integrate cartilage ECM and to promote MSC recruitment, resulting in cartilage defect repair (Liu et al., 2017; Zhang et al., 2021). The association of scaffolds with EVs and MSCs is also an alternative to improve cartilage regeneration as reported by Heirani-Tabasi *et al.* and by others (Heirani-Tabasi et al., 2021; Cho et al., 2023).

## 6 Discussion

The last 30 years of research in cartilage regeneration and its biology have been marked by numerous and essential steps forward in the technologies available to study and apply research aimed at joint regeneration. The fact that women have been a vital part of this effort, especially *via* work performed in interdisciplinary teams, is to be noted, as evidenced by the large (although non-exhaustive) body of literature cited here. It is critical for us to be at the forefront of research in a field of particular interest to us as doctors, researchers, and women (Alliston et al., 2020), but also as potential patients. If medicine has taught us anything, one must not only view one component of what we are trying to treat, but related components and how they all interact.

In the future, patients will benefit from a continuously maturing collaborative approach, combining the best biological research with robotics for biomaterial and scaffold development and printing. For example, 3D printing has already shown its relevance in creating patient-specific scaffolds based on defect evaluation *via* radiological evaluation, allowing for near-perfect replication and correction. Automated production systems for cells and cell-derived products synergize to provide relevant cells for these scaffolds in a cost effective manner. This personalized approach allows for a more precise correction and treatment, and better outcomes. Additionally, with potential for the continuous growth of the industry due to the increased incidence of OA in the general population, therapy production will be further facilitated and standardized; therefore, more patients will be able to benefit from a better access to improved technologies to treat OA.

We have also seen that OA and MSK research has diverged to genetic engineering, secretomes, and viral vectors. These approaches based on basic biological principles common to cells show promise, notably when combined with the application of stem cells. *In vitro* studies on MSC secretomes and EVs by the groups of G. Lisignoli*, of D. Noël*, and of G. J. V. M. van Osch* (van Buul et al., 2012; Manferdini et al., 2020; Ruiz et al., 2020) show promise in terms of anti-inflammatory properties and anti-catabolic effects dependent on the inflammatory status of chondrocytes and synoviocytes. These approaches allow for a more direct cellular-based approach to managing the degeneration leading to OA and the potential for slowing down the catabolic mechanisms leading to its advancement.

The future of MSK regeneration is based on a patient-centered approach. OA is a multimodal disease, with multiple factors leading to its development and progression that can be targeted to delay or even prevent its progression. The ideal scenario for future patients is a multimodal approach from personalized lifestyle modifications informed by polygenic risk scores that characterize an individual genetic risk profile as reported by the group of F. Cicuttini* (Lacaze et al., 2022) to the study of the degenerative and inflammatory state of the joint down to a cellular level to counteract destructive processes and even stimulate regeneration. Combining these different levels of therapeutic impact, one could potentially modulate the disease progression by targeting therapies to different patient phenotype in OA. There remain some challenges regarding how to make such an approach applicable, but once a multimodal method can be applied, the results may be revolutionary by targeting therapies to those most likely to benefit.

**FIGURE 2:**
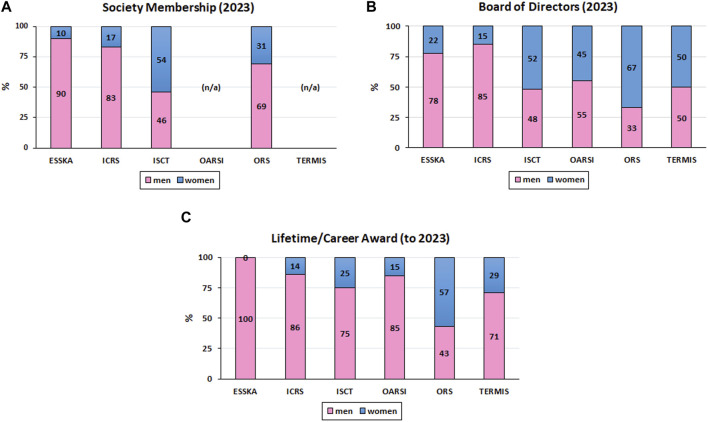
Representation of Women in MSK Societies. **(A)**: Society Membership; **(B)** Board of Directors; **(C)** Recipients of Lifetime/Career Awards; n/a: not available).

Research in the MSK field has seen many breakthroughs in the last decades, with women being at the forefront of several, especially in the field of biology. Yet, a significant disparity remains due to the generally imbalanced ratio of women to men in the field as can be noted on the representation of women in the membership (Figure 2A), in the Board of Directors (Figure 2B), and as recipients of Lifetime/Career Awards (Figure 2C) of various MSK and cell therapy/tissue regeneration societies (data generously provided by the European Society for Sports Traumatology, Knee Surgery and Arthroscopy - ESSKA - https://www.esska.org, the ICRS with a woman current President - EK*, the International Society for Cell and Gene Therapy - ISCT - https://www.isctglobal.org/home, the Osteoarthritis Research Society International - OARSI - https://www.oarsi.org, the Orthopaedic Research Society - ORS - https://www.ors.org with a woman as Past President and a woman as Incoming President, and the Tissue Engineering and Regenerative Medicine International Society - TERMIS - https://www.termis.org with a woman as Incoming President). In particular, the societies associated to clinical orthopedics (i.e., ESSKA and ICRS) are still male-dominated. Since a majority of the 2023 Board of Directors of the societies presented here are well balanced, we can speculate that this token of involvement and recognition of women in the field will translate in a better gender balance of Lifetime/Career awards in the future. The current under-representation of women in MSK research, in particular when it involves orthopedics, has a number of reasons. These include the lack of female role models and mentors in the academic world and in industry is one aspect that makes it more challenging for women to see themselves pursuing careers in MSK research. Moreover, gender prejudice and discrimination might make it more difficult for women to excel in both academia and industry, particularly in male-dominated specialties such as orthopedic surgery.

Orthopedics remains the least diversified medical specialty, with the highest prevalence of men. In fact, only 7.4% and 7% of orthopedic surgeons in the United States and UK, respectively, are female (Ahmed and Hamilton, 2021; Peterman et al., 2022). This disparity also applies in academic and research fields, with only 20% of women assistant professors, 15% of women associate professors, and 9% of women full professors in orthopedics for instance (Gerull et al., 2020), although their impact should not be overlooked. Women are also under-represented in the biomedical engineering field (Barabino et al., 2020) which is key for MSK research and translation. It is therefore crucial to take action to address these underlying problems. This can include campaigns to eliminate gender bias and discrimination as well as initiatives to raise the profile of female researchers and clinicians in academics and associated industries, such as the “Women in ESSKA” initiative that (*sic*) “wants to promote ESSKA’s educational and research activities among female orthopedic surgeons during and after their specialization” (https://www.esska.org/page/WomeninESSKA) or the “ORS Women’s Leadership Forum” that (*sic*) “will mentor, foster, encourage and inspire women at the start and throughout their careers in orthopedic research, and will assist women in obtaining leadership roles in orthopedic-related organizations” (https://www.ors.org/womens-leadership-forum).

Hope now lies in the upcoming generation of doctors and researchers, with medical and scientific Universities today having a higher prevalence of female students than males (Barabino et al., 2020; Ahmed and Hamilton, 2021). Moreover, gender should no longer be considered as binary. Hopefully, these developments will have a “butterfly” effect to increase diversity and to continue to open doors for women and minorities in the field of MSK research, including stem cell research. These new and upcoming colleagues also have the advantage of having grown up in parallel to all the new technologies of today. They have a different approach and mindset to search for opportunities, bringing a breath of fresh air and innovation to biomedical research in general. Although these newcomers may have suffered from a lack of mentorship and role models, we hope that this paper will have highlighted women who can illustrate the part and that it will inspire them to become the leaders of tomorrow in this disciplinary field (Barabino et al., 2020).
